# Evaluation of the two-step treatment with ionic liquids and alkali for enhancing enzymatic hydrolysis of *Eucalyptus*: chemical and anatomical changes

**DOI:** 10.1186/s13068-016-0578-y

**Published:** 2016-08-05

**Authors:** Han-Yin Li, Xue Chen, Chen-Zhou Wang, Shao-Ni Sun, Run-Cang Sun

**Affiliations:** 1 Beijing Key Laboratory of Lignocellulosic Chemistry, Beijing Forestry University, Beijing, 100083 China; 2 State Key Laboratory of Pulp and Paper Engineering, South China University of Technology, Guangzhou, 510640 China

**Keywords:** *Eucalyptus*, Ionic liquids pretreatment, Alkali fractionation, Enzymatic hydrolysis, Anatomical changes

## Abstract

**Background:**

The biomass recalcitrance resulting from its chemical compositions and physical structures impedes the conversion of biomass into fermentable sugars. Pretreatment is a necessary procedure to increase the cellulase accessibility for bioconversion of lignocelluloses into bioethanol. Alternatively, ionic liquids, a series of promising solvents, provide unique opportunities for pretreating a wide range of lignocellulosic materials. In this study, a two-step treatment including ionic liquids pretreatment and successive alkali fractionations was performed on *Eucalyptus* to achieve a high enzymatic digestibility. The compositional and structural changes of *Eucalyptus* cell walls and their possible effect on saccharification ratio were comprehensively investigated.

**Results:**

After the ionic liquids pretreatment, the cell walls became loose and even swelled, accompanying with the decrease of cellulose crystallinity. As compared to the simplex ionic liquids pretreatment, the integrated process resulted in the significant removal of hemicelluloses and lignin, enhancing the disruption of the cell walls and increasing the exposure of cellulose, which led to a higher conversion of cellulose to glucose. The glucose yield of *Eucalyptus* underwent the combination of [Bmim]OAc and alkali treatments reached the maximum (90.53 %), which was 6.6 times higher than that of the untreated *Eucalyptus*. The combination of chemical compositions and physical structure of *Eucalyptus* affected the efficiency of cellulose enzymatic hydrolysis. Especially, the changes of cellulose crystallinity played a major role in enhancing the enzymatic digestibility of *Eucalyptus* in this study.

**Conclusions:**

The two-step treatment with ionic liquids pretreatment and successive alkali fractionation can be considered as a promising method to improve the conversion of cellulose to glucose. The detailed information obtained about chemical and anatomical changes was helpful to understand the underlying mechanism of the integrated treatment process acting on *Eucalyptus* for enhancing enzymatic digestibility.

**Electronic supplementary material:**

The online version of this article (doi:10.1186/s13068-016-0578-y) contains supplementary material, which is available to authorized users.

## Background

Lignocellulosic biomass, the most abundant renewable energy source on the earth, is a promising biofuel resource for replacing fossil fuels [1, 2]. The main constituents of lignocellulosic biomass are cellulose, hemicelluloses, and lignin. The cellulose microfibril framework is tethered together by a coating of hemicelluloses and sealed in a polymeric matrix of lignin, forming a rigid and compact structure, which restricted the conversion of cellulose into fermentable sugars for bioethanol production [3, 4]. The presence of hemicelluloses, lignin and their spatial inter-links constructs the physical barriers of biomass. Additionally, cellulose crystallinity, the presence of acetyl groups, the degree of polymerization, and specific surface area also limit the enzymatic hydrolysis of biomass [5, 6]. The chemical compositions and physical structures of biomass constitute the recalcitrance for preventing cellulose from degradation, while increasing the conversion costs. Therefore, pretreatment is necessary to reduce the resistance and make biomass or cellulose highly digestible to enzymatic attack.

During the last few decades, a number of pretreatment methods have been developed. Among these pretreatments, ionic liquids (ILs) have emerged as promising solvents for the dissolution of lignocellulosic biomass in recent years [7]. ILs are salts, typically composed of a large organic cation and a small anion [8]. Unlike traditional organic solvents, ILs have many unique properties such as non-flammability, low vapor pressure, wide liquid range, high chemical and thermal stability, and good solvating properties [9, 10]. It has been reported that ILs have the ability of dissolving biopolymers, especially cellulose, and reducing the crystallinity of cellulose, thus enhancing the enzymatic hydrolysis of cellulose [11, 12]. Actually, the physical and chemical properties of ILs can be varied by adjusting their anions and cations, thus different ILs resulted in a wide range of carbohydrate solubilities. Additionally, one of the main advantages of using ILs to dissolve carbohydrates is that ILs can be tailored based on the needs of a specific dissolution or functionalization of these polymers [13, 14]. Though some changes of chemical composition could occur during the ILs pretreatment, more lignin and hemicelluloses should be removed for achieving a higher enzymatic hydrolysis efficiency. Therefore, a further treatment needs to be implemented to remove more hemicelluloses and lignin from the pretreated substrates. Alkali treatment has been widely used to effectively remove lignin and partial hemicelluloses, to disrupt the rigid structure of biomass, and to increase the porosity and surface area, thereby increasing the enzymatic hydrolysis [15]. Among various alkalis, NaOH can effectively break the linkages between lignin units, or between lignin and hemicelluloses, particularly ether and ester bonds, and cause the cell wall swelling, the decrease of the degree of polymerization and crystallinity, and the increase of the internal surface of cellulose [15, 16].

Biomass recalcitrance is a multi-scale phenomenon involving several orders of magnitude containing both macroscopic and microscopic barriers [17]. Various pretreatments enhance the digestibility of biomass by different mechanisms. Therefore, although much is known about the ILs and alkali treatments acting on biomass at macro-level performance in recent years, more information about how the microstructure and even nanostructure of plant cell wall respond to the pretreatment should be obtained for enhancing the understanding of the pretreatment mechanisms, and then optimizing the pretreatment process. In the present study, a two-step treatment process composed of ILs and successive alkali post-treatment was applied to *Eucalyptus* for improving the enzymatic saccharification. To understand the relationship in the changes of chemical compositions, physicochemical characteristics, morphology, and topochemistry occurring in *Eucalyptus* during the treatments for improving the enzymatic digestibility, the ILs-pretreated and alkali post-treated samples were detected by high-performance anion exchange chromatography (HPAEC), Fourier transform infrared (FT-IR), X-ray diffraction (XRD), scanning electron microscopy (SEM), confocal Raman microscopy (CRM), and atomic force microscopy (AFM).

## Results and discussion

### Morphological and topochemical changes in *Eucalyptus* cell walls

**Fig. 1:**
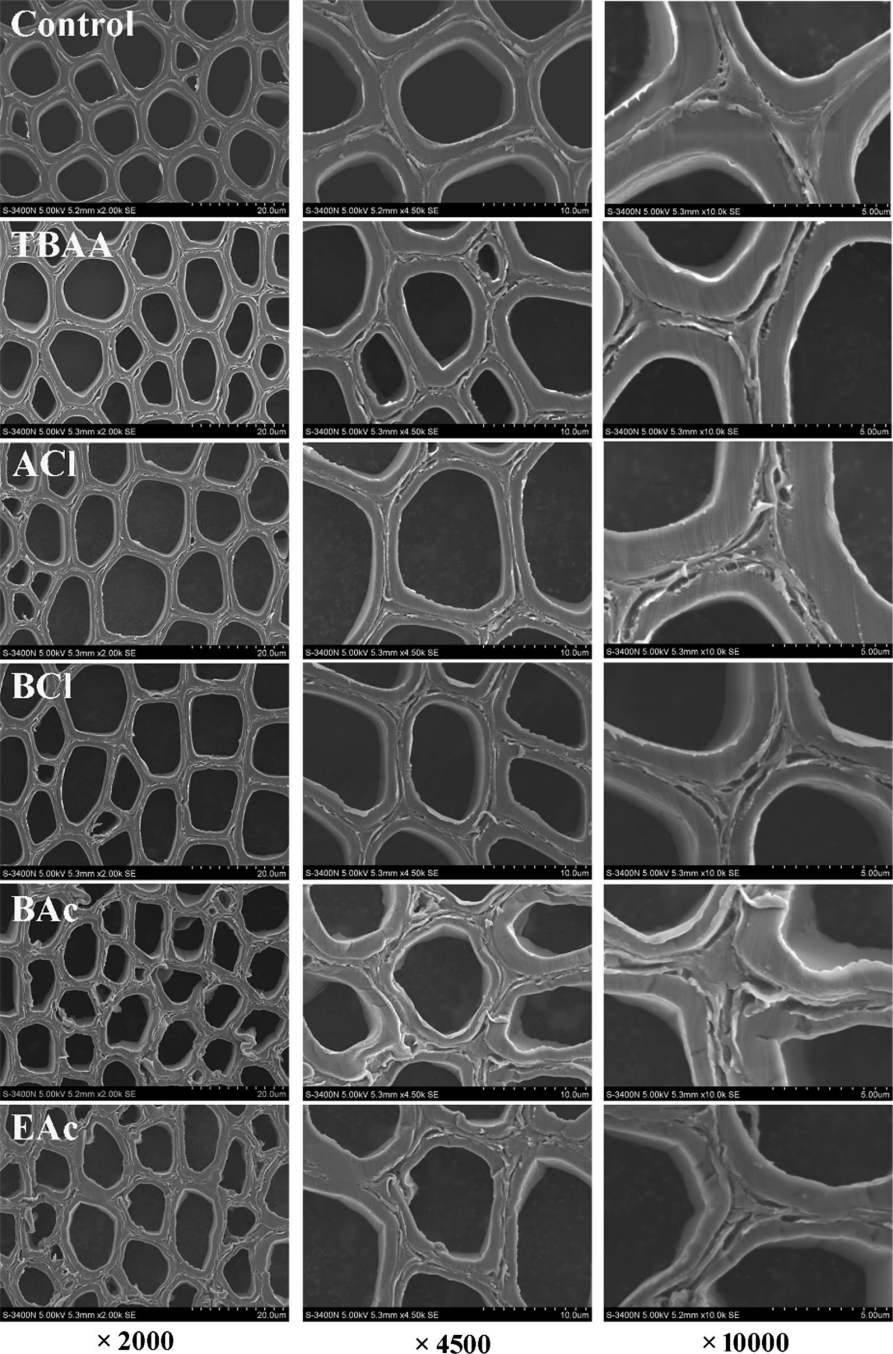
SEM images of the control and ILs-pretreated *Eucalyptus* at magnification ×2000, ×4500, and ×10,000

**Fig. 2:**
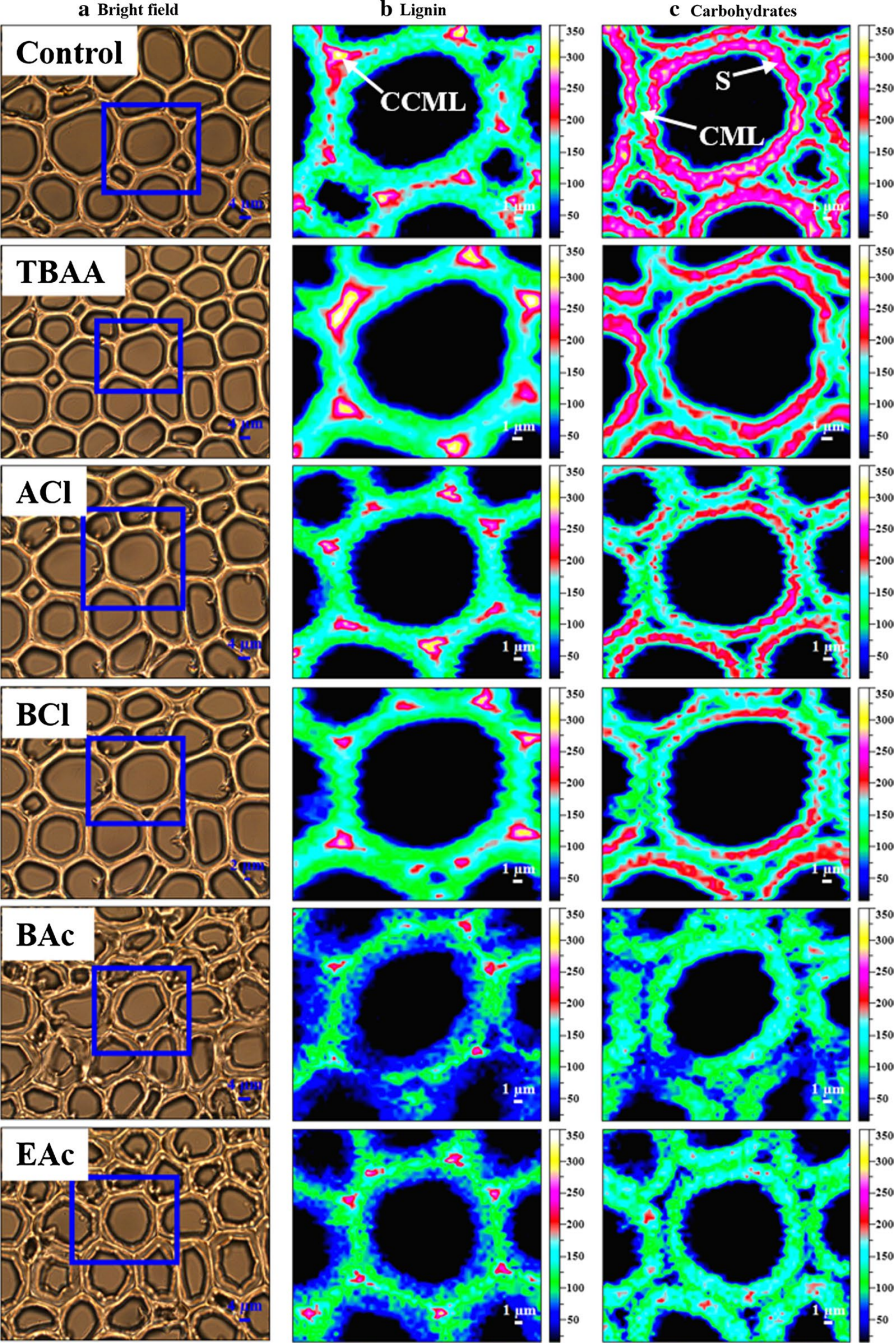
Bright field images of the cell wall of the control and ILs-pretreated *Eucalyptus* (**a**). Selected areas (*blue rectangle*) shown in bright field images were used for Raman imaging. Raman images of the main components distribution in the *Eucalyptus* cell walls before (control) and after each ILs pretreatment by integrating from 1560 to 1625 cm^−1^ (**b**) and from 2830 to 2920 cm^−1^ (**c**)

The SEM images of the untreated and ILs-pretreated *Eucalyptus* taken at various magnifications are shown in Fig. 1. It was observed that the raw material had a highly ordered structure and compact morphology with trace evidence of mechanical damage from the cutting process. The morphological changes in the pretreated samples were various depending on the different ILs. After the five ILs pretreatments, the cell wall structures became loose as the cracks appeared between the adjacent cell walls, which were more clearly visible in the higher magnification images. The formation of cracks might be associated with the partial delignification during the ILs pretreatment process. More disruptions such as disorder and distortion were observed in the cell walls pretreated with [Bmim]OAc and [Emim]OAc. These morphological changes in the samples pretreated with the five ILs were not found in the bright field images (Fig. 2a). The fact may be explained that the swelling of the cell walls occurred during the ILs pretreatment, thus causing the shrinkage of the swelled cell walls during the drying process [18].

The visualized information about the compositional distribution of *Eucalyptus* cell walls pretreated with five different ILs at subcellular level was obtained in situ by CRM. Figure 2a shows the bright field images of the samples. As can be seen, the cell walls in the untreated section which were intact and highly compact underwent different degrees of variation after the ILs pretreatment. Almost no changes appeared in the cell walls pretreated with TBAA. The cell walls were slightly broken during the [Amim]Cl and [Bmim]Cl pretreatments, while significant changes occurred in the cell walls pretreated with [Bmim]OAc and [Emim]OAc. Especially, the cell walls of BAc and EAc were swollen and their structures were disordered and even distorted as compared with the cell walls untreated and pretreated with the other three ILs.

The selected areas (blue rectangle) shown in bright field images (Fig. 2a) were used for Raman imaging, and the two-dimensional chemical images were acquired by integrating over the characteristic Raman bands. The regions of 1560–1625 cm^−1^ dominated by the contribution of symmetric stretching of the aromatic ring were used to generate the images of lignin distribution [19]. The prominent bands of carbohydrates can be easily detected in 2830–2920 cm^−1^ known for the C–H and C–H_2_ stretching [20, 21]. The Raman images of lignin and carbohydrates distributions in the untreated and ILs-pretreated fiber cell walls are shown in Fig. 2b and c, respectively. It was found that the raw material had a heterogeneous distribution of the components within morphologically distinct regions as the various intensity reflected the different concentrations. Clearly, a high intensity of lignin was observed in the cell corner middle lamella (CCML) regions, followed by compound middle lamella (CML), and the lowest in the secondary wall (S) regions. However, the highest concentration of carbohydrates in the untreated cell wall appeared in the S regions and the lowest in the CCML regions. After the ILs pretreatment, the concentrations of the main compositions in these pretreated samples varied with IL type. As compared with the untreated sample, the pretreatment with TBAA resulted in a slight reduction of carbohydrates and lignin. The two samples after pretreated with [Amim]Cl and [Bmim]Cl had a similar decreasing trend in the concentrations of carbohydrates and lignin, which had a comparatively more reduction than that in the TBAA-treated sections. The intensities of lignin and carbohydrates became weaker in all the morphologically distinct cell wall regions after the pretreatment with [Emim]OAc, and the most significant delignification and dissolution of carbohydrates were observed in the sample pretreated with [Bmim]OAc. The phenomenon may be due to the fact that the swelling of cell wall is very important for transporting lignin fragments out of the cell wall in delignification, as well as for the dissolution of cellulose and hemicelluloses [22, 23].

**Fig. 3:**
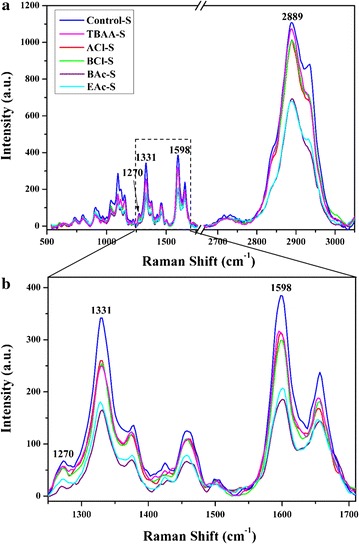
Average Raman spectra of S regions in the cross sections of the control and ILs-pretreated *Eucalyptus* (**a**), and zoom into the average Raman spectra in the range of 1250–1710 cm^−1^ (**b**)

To analyze the detailed changes from the small distinct morphological regions after ILs pretreatment, average Raman spectra were extracted from the S regions which accounted for a large portion of the cell walls as shown in Fig. 3. It was found that all Raman signal intensities in the substrates pretreated with different ILs decreased in varying degrees. The characteristic band intensities of lignin and carbohydrates at 1598 and 2889 cm^−1^, respectively, showed a reduction in the sample of TBAA as compared with that in the untreated sample, indicating the removal of lignin and carbohydrates. A similar performance in delignification and more dissolution of carbohydrates were observed after the pretreatment with [Amim]Cl and [Bmim]Cl. It should be noted that a great loss of the both lignin and carbohydrates occurred during the pretreatment with [Bmim]OAc and [Emim]OAc, and the lowest band intensity of lignin was observed in the sample of BAc, suggesting the best effect on the delignification.

Lignin is a complex and heterogeneous biopolymer composed of syringyl (S), guaiacyl (G), and *p*-hydroxyphenyl (H) units. It has been reported that the ratio of S/G is connected with the recalcitrance of untreated biomass, which affects the cell wall deconstruction during the chemical pretreatment, and the sequential hydrolysis of cellulose to glucose [24–26]. Raman spectroscopy technology as a rapid and non-destructive method has been used in detecting the S/G ratio of lignin, and the characteristic Raman bands at 1331 and 1270 cm^−1^ corresponding to the *S* and *G* units, respectively, were selected to monitor the changes of the two units [27–29]. As can be seen from Fig. 3b, the *S* bands showed much stronger intensities than the *G* peaks in the untreated sample. After the ILs pretreatment, the intensities of *S* and *G* bands decreased, similar to the corresponding lignin bands, suggesting that ILs pretreatment is very effective on the depolymerization of both *S* and *G* units. To analyze the changes of *S*/*G* ratios in the *S* layers of *Eucalyptus* cell wall pretreated with five different ILs, the spectral deconvolution was performed in the range of 1250–1530 cm^−1^ according to a previous literature [27]. The intensity ratio of the resolved *S* to *G* bands which has been confirmed to have a linear relationship with the corresponding molecular ratio was used to evaluate the *S*/*G* ratio [27]. Therefore, Raman spectroscopy technique was used for trend analysis of *S*/*G* ratio changes in this study. The intensities of the resolved *S* and *G* bands and their ratios are shown in Additional file 1: Figure S1. As can be seen, the samples pretreated with [Bmim]OAc and [Bmim]Cl showed the highest and lowest *S*/*G* ratio, respectively, as compared to the other substrates. Generally, *S*/*G* ratio could affect the crosslinking between lignin and other cell wall components and the microscopic structure and topochemistry of plant cell wall, thus further influencing the enzyme accessibility [26, 30]. It has been reported that the sample with a high *S*/*G* ratio is easier to be pretreated owing to the existence of a higher proportion of labile *β*-*O*-4 bonds in lignin [31]. In this case, the pretreatments are able to release cellulose, thus increasing the sugar yields after enzymatic hydrolysis [25, 31].

Based on the fact shown in Figs. 2 and 3, the ILs contained the same [Bmim] cation but different anions showed the varied effects on the structure and composition of *Eucalyptus* cell walls, while the ILs with the same acetate or chloride anion had the similar performances, indicating that the role of anion was predominant as compared to the contribution from cation [32]. The reason for this was that the hydrogen-bonding interactions between the anion of ILs and hydroxyl protons of cellulose would be much stronger than those of the hydroxyl oxygen and ether oxygen of cellulose with protons on the IL cations [32]. Better performances in swelling and delignification of cell walls were obtained by the acetate ILs than the chloride ILs. It has been reported that the acetate anion with a strong basicity made it more efficient in disrupting the inter- and intra-molecular hydrogen bonding in biomass than chloride anion, since ILs with a strong hydrogen bond basicity meaning a strong hydrogen bond acceptor capacity can effectively weaken the hydrogen-bonding network in the polymer chains [33, 34]. In this study, the effect of [Bmim]OAc was slightly superior to [Emim]OAc, which was contrary to the previous research that the smaller sized [Emim] cation might be more effective than [Bmim] cation in the pretreatment of rice straw owing to the greater interaction of [Emim] cation with the cellulose chain [35]. This discrepancy may be caused by the difference in the sample species and treatment conditions.

### Chemical composition

Based on the results of the above section, the samples pretreated with [Bmim]Cl (mild) and [Bmim]OAc (severe) having the same cation were selected as representatives for further investigated. The two pretreated samples BCl and BAc were treated with conventional successive alkali treatment to extract more lignin and hemicelluloses, and then they were undergone comprehensive analysis to elucidate the origins of the differences in treatment result.

The chemical compositions of the untreated and treated *Eucalyptus* are given in Table 1. Obviously, no considerable changes in the composition of the samples were observed as a result of the ILs pretreatment. After the [Bmim]OAc pretreatment, it was observed that only 6.36 % of the total mass were consumed. The low reduced biomass recovery was mainly attributed to the removal of lignin, since 16.97 % of total lignin were removed during the [Bmim]OAc treatment process corresponding to 71.08 % of the mass loss. The lignin solubilization during the ILs pretreatment has been reported to be assisted by the *π*–*π* interactions of the IL cation with lignin [36]. A more effective delignification was reported that 15.1 % of the total mass was lost and 32.1 % of the initial lignin was removed from energy cane bagasse by [Emim]OAc at 120 °C for 30 min [12]. Additionally, nearly 50 % of the lignin extraction from the maple wood flour was reached with [Bmim]OAc at 90 °C for 24 h [37]. The lower lignin loss in this study than those in the previous studies can be stemmed from the short pretreatment time and the high density of *Eucalyptus*, which was because a longer pretreatment time could improve the dissolution capacity of biomass. However, the high density *Eucalyptus* had a tighter structure than energy cane bagasse, which resulted in a more difficult access of ILs to raise the pretreatment efficiency [13, 38]. Xylan, the dominant carbohydrates in *Eucalyptus* hemicelluloses, showed a minor reduction after the [Bmim]OAc pretreatment. Therefore, the slight increase of the relative content of glucan from 41.58 to 43.94 % after the ILs pretreatment was mainly due to the removal of lignin and small amounts of hemicelluloses. Obviously, the reduction of lignin and hemicelluloses in BCl was lower than that in BAc. This was in line with the above result of Raman analysis, in which the acetate ILs were more effective in cell walls swelling and delignification as compared to the chloride ILs.

**Table 1:** Chemical compositions of the control, ILs-pretreated, and alkali post-treated substrates obtained after the synergistic treatment

Samples^a^	Glucan (%)		Xylan (%)		AIL (%)	ASL (%)	Lignin (%)		Solid recovery (%)
	*C*	*R*	*C*	*R*			*C*	*R*	
Control	41.58		15.85		25.44	3.96	29.40		100
BCl	42.32	0.57	15.87	2.18	23.77	4.07	27.84	7.50	97.69
BAc	43.94	1.05	14.84	12.33	21.81	4.26	26.07	16.97	93.64
Control-4	46.99	10.49	12.17	39.18	25.20	3.88	29.08	21.65	79.21
BCl-4	47.94	11.32	12.24	40.60	24.02	3.66	27.68	27.58	76.92
BAc-4	48.90	11.99	11.94	43.63	23.09	3.81	26.90	31.53	74.83

^a^Control: raw material; BCl and BAc: samples obtained after the pretreatment with [Bmim]Cl and [Bmim]OAc at 120 °C for 30 min, respectively; Control-4, BCl-4, and BAc-4: residues obtained after the successive treatment with 0.5, 2.0, and 4.0 % NaOH at 90 °C for 2 h from the untreated, [Bmim]Cl-pretreated, and [Bmim]OAc-pretreated *Eucalyptus*, respectively

*AIL* acid insoluble lignin, *ASL* acid soluble lignin, *C* the content of each composition, *R* the removal of each composition after treatment on the basis of its original amount in the raw material

By comparing with the single ILs-pretreated samples, a greater mass loss was observed after the alkali post-treatment. It has been found that some alkali-labile linkages in lignin units, or between lignin and carbohydrates were cleaved during the alkali treatment, resulting in the dissolution of lignin and hemicelluloses [39]. Obviously, the mass loss of the ILs-pretreated samples was more than that of the raw material after the alkali treatment, which was probably caused by more liberation of reactive sites and accessible area in the raw material during the ILs pretreatment. It should be noted that the loss of xylan (39.18–43.63 %) was enhanced significantly by the alkali treatment as evidenced by the decrease of its relative contents, which was higher than that of lignin (21.65–31.53 %), indicating that the increase of the relative content of glucan was mainly attributed to the dissolution of hemicelluloses. Based on the above results, the synergistic effect of ILs and alkali treatments could result in more removal of hemicelluloses and lignin than the ILs pretreatment alone, thus leading to the increment of the relative content of glucan.

### Physicochemical characteristics of the untreated and treated samples

The structural changes of *Eucalyptus* after the treatments were investigated by FT-IR, and the spectra are shown in Additional file 1: Figure S2. It should be noted that the spectra generated by the samples treated with ILs or alkali were similar to that of the raw materials except a great discrepancy observed at 1737 cm^−1^. The intensity of the band at 1737 cm^−1^ corresponding to carbonyl groups from hemicelluloses was decreased in BCl and BAc, indicating the removal of hemicelluloses as shown in Table 1 [40]. However, the peak at 1737 cm^−1^ disappeared in all the samples treated with alkali, confirming that the ester bands especially acetyl groups in hemicelluloses were cleaved significantly by the alkali treatment [41, 42]. As expected, the acetyl groups existed in hemicelluloses were considered as one of the barriers for the biodegradation of lignocellulosic biomass [43]. Therefore, the extensive deacetylation in hemicelluloses after the alkali treatment may contribute to improving the enzymatic digestibility of biomass [44]. Additionally, the slight decreases of the bands at 1503 cm^−1^ (aromatic skeletal vibration of lignin) and 1325 cm^−1^ (*S* and condensed *G* absorptions) were observed in the ILs-pretreated as well as alkali-treated samples, suggesting that the delignification of biomass occurred both in the ILs pretreatment and alkali fractionation [45]. Furthermore, the intensities of the peaks at 1369 cm^−1^ (C–H deformation in cellulose and hemicelluloses), 1235 cm^−1^ (C–O stretching in lignin and hemicelluloses), and 1027 cm^−1^ (C–O stretch in cellulose and hemicelluloses) decreased likely owing to the removal of hemicelluloses, which was in line with the results of chemical composition analysis [46]. The –CH_2_ scissoring motion band at 1422 cm^−1^ (strong in type I crystalline) had the weakest intensity in the sample pretreated with [Bmim]OAc, followed by [Bmim]Cl, as compared to the untreated raw materials [47, 48]. Conversely, an increase of the intensity was observed at 899 cm^−1^ attributed to anti-symmetric out-of-plane ring stretch of amorphous cellulose [49]. The fact suggested that the decrease of cellulose crystallinity occurred during the ILs pretreatment.

**Fig. 4:**
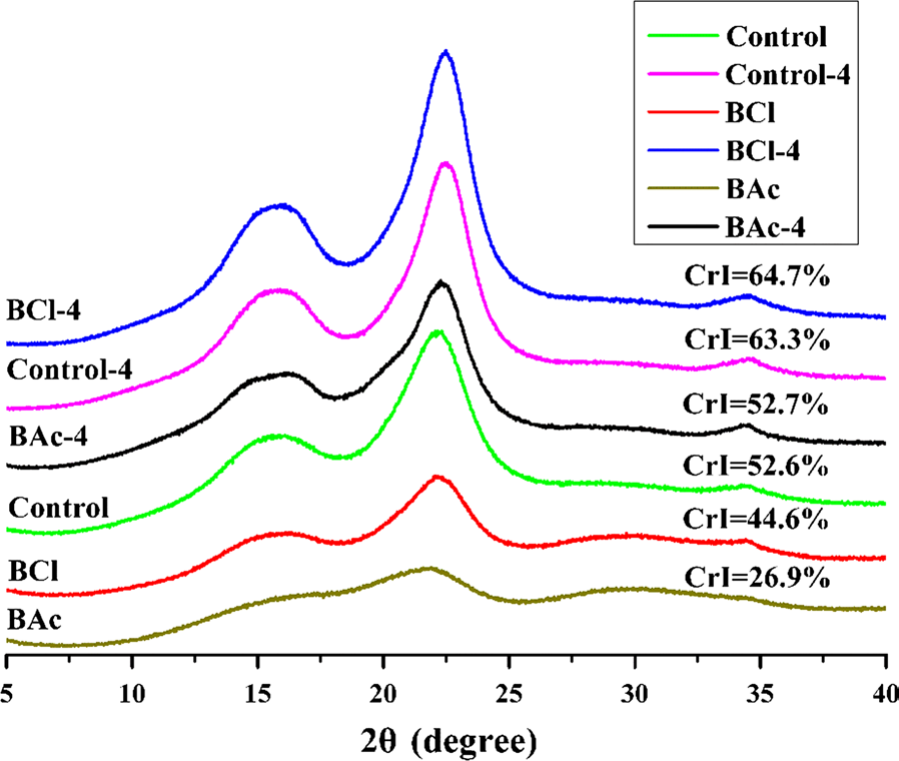
XRD spectra of the control, ILs-pretreated, and alkali post-treated substrates

As is known that the crystalline region in the cell walls of biomass could impede the cellulases accessing to cellulose, resulting in a low cellulose saccharification ratio [50]. To further study the impact of the ILs pretreatment and alkali post-treatment on the crystal structure of cellulose in *Eucalyptus*, the crystallinity index (CrI) of the untreated and treated substrates was calculated by XRD as shown in Fig. 4. In this study, the two typical diffraction peaks appeared at 2*θ* = 15.8° and 22.3°, corresponding to (101) and (002) lattice planes of crystalline cellulose I, respectively [12]. It was obvious that the XRD patterns of the samples obtained after ILs pretreatment were changed significantly and appeared a broad diffraction peak, and the peak at 15.8° almost disappeared in BAc. As expected, the minimum value of CrI was obtained in BAc (26.86 %), indicating the lowest crystallinity, followed by BCl (44.56 %). Thus, the ionic liquid [Bmim]OAc turned out to be more effective than [Bmim]Cl in reducing the crystallinity of *Eucalyptus*, which was in accordance with the FT-IR analysis. Generally, during the ILs pretreatment, some of the inter- and intra-molecular hydrogen bonds and the crystalline structure of cellulose in the raw material were destroyed, which resulted in a more amorphous structure, thus increasing the cellulose surface accessibility and theoretically improving the efficiency of enzymatic hydrolysis [46, 51]. However, the increase of CrI in the alkali-treated samples as compared to the ILs-pretreated *Eucalyptus* was observed, which was mainly attributed to the removal of some amorphous components such as xylan and lignin [52].

### Morphological analysis

**Fig. 5:**
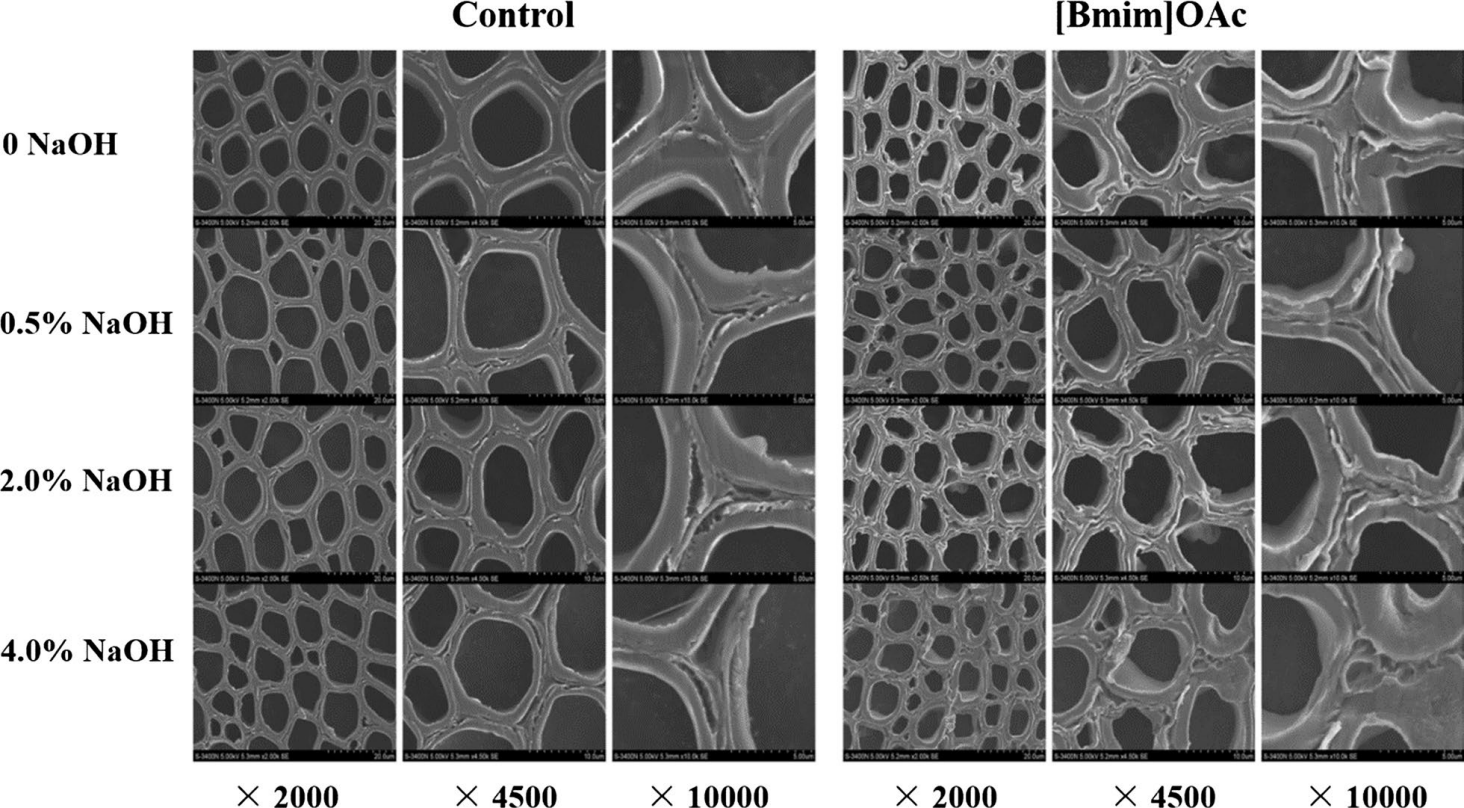
SEM images of the control, [Bmim]OAc-pretreated, and alkali post-treated *Eucalyptus* at magnification ×2000, ×4500, and ×10,000

SEM images of control, [Bmim]OAc-pretreated, and alkali post-treated *Eucalyptus* are shown in Fig. 5. Obviously, the raw material displayed a rigid and highly ordered structure, which hindered the accessibility of cellulase to cellulose. After the simple alkali treatment, the compact structure of cell wall was disrupted with formation of cracks, especially at the CCML regions. As is known to all, lignin is essential to the structural integrity of cell walls and rich in the CCML regions, while hemicelluloses are considered as an adhesive in the cell walls and enriched in the boundary of layers [53, 54]. Therefore, the disruption of the cell walls may be caused by the removal of hemicelluloses and lignin. As the increment of alkali concentration from 0.5 to 4.0 %, the degree of damage was increasing gradually as evidenced by the expanding crevices. When the NaOH concentration reached 4.0 %, a part of CCML almost disappeared, which may also be related to the delignification in the cell walls. The synergetic treatment opened up the cell wall structure and resulted in an enhanced surface area for enzymes to attach cellulose.

**Fig. 6:**
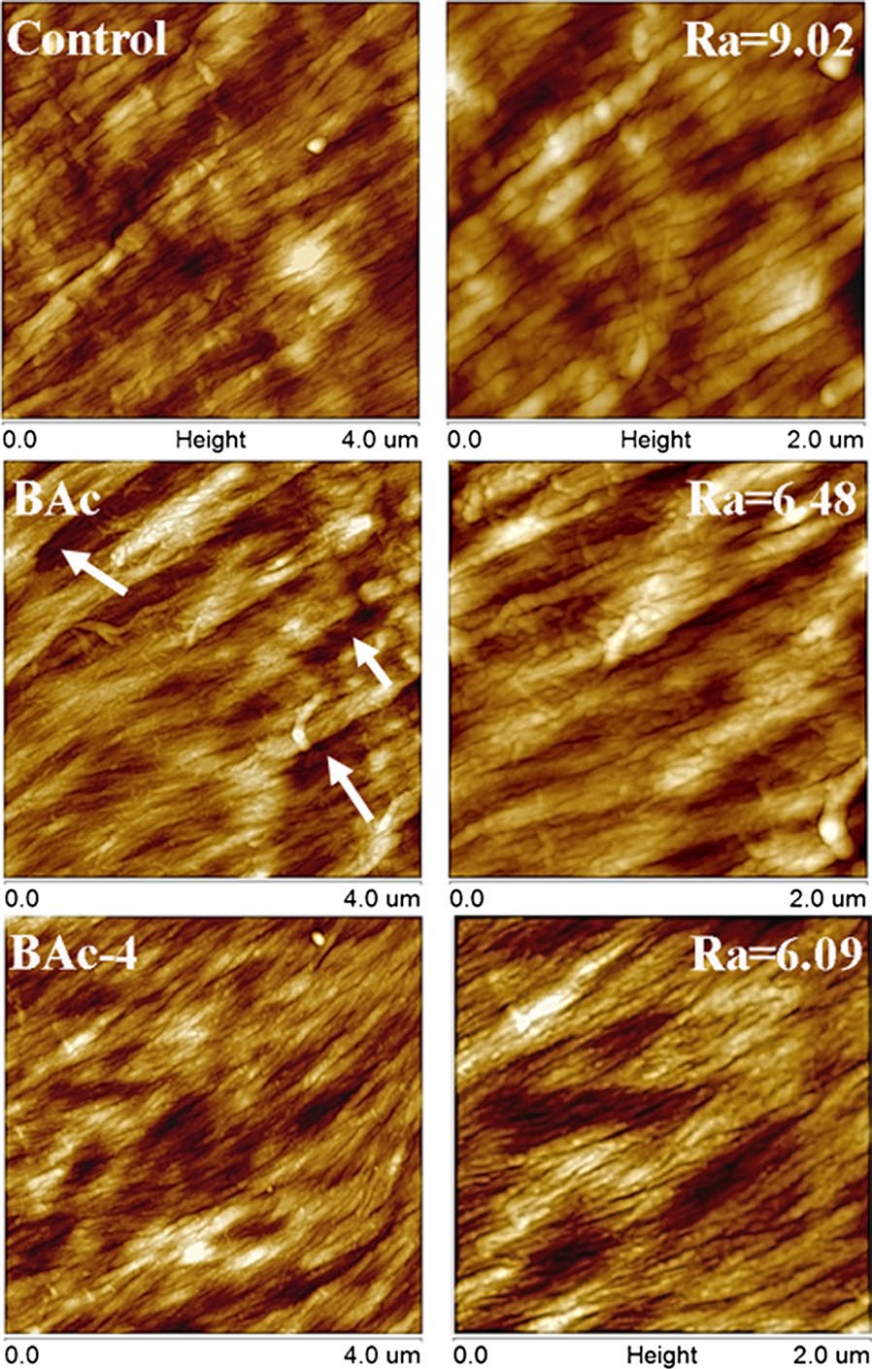
AFM height images of the control, [Bmim]OAc-pretreated, and [Bmim]OAc-alkali-treated *Eucalyptus* fiber cell wall from S layers at different magnifications. The *white arrows* point out the cracks between microfibrils after [Bmim]OAc pretreatment

AFM has become a powerful tool in characterization of surface structure and morphology of lignocellulosic biomass. The AFM height images of the untreated, [Bmim]OAc-pretreated and [Bmim]OAc-alkali-treated *Eucalyptus* fiber cell wall in *S* layers are shown in Fig. 6, because the *S* layer accounts for the largest proportion and it is the most predominant layer in cell walls. It was observed that the microfibrils in the untreated cell walls had a specific orientation and rough surface structure. The rough surface structure may be attributed to the fact that lignin usually formed aggregates and existed on the cellulose microfibril surface in a grainy shape [55]. In addition, hemicelluloses were mainly distributed between lignin and cellulose and coated on the cellulose microfibril surface [55]. In other words, cellulose microfibrils as the cell wall scaffold were embedded in a matrix of hemicelluloses and phenolics substances, resulting in a compact and rigid structure [56]. After the [Bmim]OAc pretreatment, some cracks appeared between the microfibrils, which could increase the surface area and promote the disruption of lignocelluloses. Furthermore, it was found that the surface roughness Ra decreased dramatically from 9.02 to 6.48. These facts may be related to the removal of matrix polymers, as evidenced by the composition analysis and Raman images. However, after the subsequent alkali treatments, the cellulose microfibril surface became smoother by removing more hemicelluloses and lignin, thus enhancing the exposure of cellulose microfibrils without substantially altering the microfibril orientation and arrangement. These changes in the ultrastructure of cell wall can effectively open up the cell wall structure and expose cellulose fibrils for increasing the accessibility and available binding sites of enzyme.

### Enzymatic hydrolysis

**Fig. 7:**
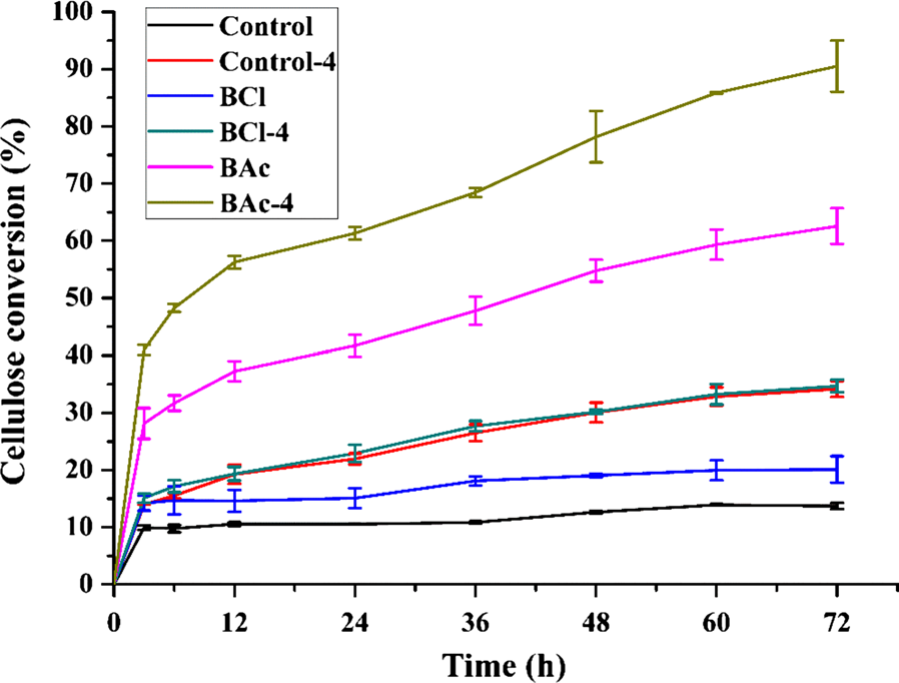
The enzymatic hydrolysis of the control, ILs-pretreated, and alkali post-treated substrates. The *error bars* are standard deviations from the average values of duplicate determinations

Enzymatic digestibility is closely related to the treatment effectiveness and is an outstanding probe for the accessibility of cellulose [56]. Figure 7 shows the glucose released from the enzymatic hydrolysis of the raw material and after treated samples. As expected, the cellulose conversion generally increased with the incubation time. After 72 h, enzymatic hydrolysis led to 13.71 % of glucan converting into glucose in the untreated *Eucalyptus*. The difficulty of enzymatic hydrolysis in untreated lignocellulosic biomasses was attributed to the presence of hemicelluloses and lignin and their spatial inter-links, which built the physical barriers to protect the cellulose from degradation [1, 57]. After the ILs pretreatment, the faster saccharification rates as well as higher sugar yields were achieved, especially for the sample pretreated with [Bmim]OAc, in which a glucose yield of 62.56 % was obtained. The reason for the large discrepancy of cellulose conversion between the BCl and BAc may be attributed to the lower CrI value of BAc, which was in agreement with the previous literature [11]. The glucose yield of Control-4 treated with alkali alone was facilitated by removal of matrix polymers as compared to the raw material, while it was lower than that of BAc which showed less degree of delignification. The phenomenon was presumed that the removal of matrix polymers was helpful to increase the efficiency of enzymatic hydrolysis, while it was not a prerequisite for raising the accessibility of cellulose [5]. The outstanding pretreatment performance of [Bmim]OAc mainly stemmed from the significant changes in cellulose crystallinity, instead of delignification. Apparently, the alkali post-treatment which was more effective to remove hemicelluloses and lignin led to a substantial improvement in enzymatic hydrolysis as compared to the corresponding ILs pretreatment. This could be explained by the fact that more removal of lignin and hemicelluloses would expose a larger fiber surface area for the enzymes and reduce the useless adsorption of cellulase on lignin, suggesting that the subsequent alkali treatment was useful for increasing the conversion of cellulose to glucose. It was noted that the sample suffered from the combination of [Bmim]OAc pretreatment and alkali treatment achieved the maximum glucose yield (90.53 %), which was about 6.6 times higher than that of the untreated *Eucalyptus*. Both a significant increment in the amorphous cellulose by [Bmim]OAc pretreatment and the removal of hemicelluloses and lignin by alkali extraction synergistically resulted in a high cellulose conversion. However, there was no need to remove the majority of lignin for achieving more than 90 % glucose yield, as only 31.53 % of total lignin were removed by the treatment combined [Bmim]OAc and alkali based on the chemical composition analysis. These results demonstrated that the chemical compositions and physical structure of biomass synergistically affected the accessibility of cellulose. Though the delignification was critical for reducing the barrier of enzymatic hydrolysis, the disruption of the cellulose crystallinity was even more vital to increasing the cellulose surface area and made the biomass more digestible to the cellulases in this study.

## Conclusions

The comprehensive information that the effect of ILs pretreatment combined with alkali treatment on the enzymatic hydrolysis was obtained by the wet chemical analysis and microscopic measurements. The results indicated that the ILs pretreatment did not substantially alter the chemical composition of biomass, but did change their structural features such as the swelling of cell walls and the decline of cellulose crystallinity. The alkali treatment could largely remove hemicelluloses and partly reduce the content of lignin, thus disrupting the structure of cell walls. Both the ILs pretreatment and alkali fractionation could enhance the enzymatic digestibility of cellulose in a certain extent. The combined treatment of ILs and alkali was the most effective approach for increasing the efficiency of enzymatic hydrolysis as compared to the ILs or alkali treatment alone, owing to the high degree removal of matrix polymers, significantly disruption of cell walls, generation of more amorphous cellulose structure, and the exposure of cellulose microfibrils. The chemical compositions and physical structure of plant biomass had a synergistic effect on the enzymatic hydrolysis process. Actually, the role of physical structure was more critical than chemical compositions in this study.

## Methods

### Raw materials

*Eucalyptusgrandis* × *E. urophylla* wood (5 years old) was the raw material in this study obtained from Guangxi province, China. The dried raw material was ground, and the particles (80–100 mesh) were first extracted with methylbenzene–ethanol (2:1, v/v) in a Soxhlet apparatus for 6 h, and then were dried for 16 h in an oven at 60 °C for further use. For microscopic measurements, 8 μm thickness cross sections were cut from the raw material by a sliding microtome. The sections were then used for ILs pretreatment and alkali fractionation. The different types of ILs, such as tetrabutylammonium acetate (TBAA), 1-allyl-3-methylimidazolium chloride ([Amim]Cl), 1-butyl-3-methylimidazolium chloride ([Bmim]Cl), 1-butyl-3-methylimidazolium acetate ([Bmim]OAc), and 1-ethyl-3-methylimidazolium acetate ([Emim]OAc), were purchased from Lanzhou Institute of Chemical Physics, Lanzhou, China. All chemicals purchased were of analytical or reagent grade and used without further purification.

### ILs pretreatment and alkali fractionation

The dewaxed *Eucalyptus* samples (5 g) were pretreated with TBAA, [Amim]Cl, [Bmim]Cl, [Bmim]OAc, and [Emim]OAc in a 250 mL flask at a biomass loading of 5 wt%, respectively. The mixtures were heated in an oil bath at 120 °C for 30 min with magnetic stirring. After treatment for the designated time, quintuple deionized water as an antisolvent was slowly added into the ILs solution with constant stirring for 30 min at room temperature. Subsequently, the suspensions were filtered to separate the pretreated wood. The solids were washed repeatedly with deionized water to remove any remaining ILs from the samples until the wash solution appeared colorless. Finally, the solids were dried at 60 °C for 24 h and weighed to determine the recovery of solid obtained after pretreatment. The ILs-pretreated samples were labeled as TBAA, ACl, BCl, BAc, and EAc, respectively, according to the used ILs as TBAA, [Amim]Cl, [Bmim]Cl, [Bmim]OAc, and [Emim]OAc. In alkali fractionation, the [Bmim]Cl and [Bmim]OAc-pretreated samples were further successively treated with 0.5, 2.0, and 4.0 % NaOH at 90 °C for 2 h under a solid to liquid ratio of 1:20 (g/mL). The insoluble residues in each step were collected by filtration, thoroughly washed with deionized water and dried, and the last residues were labeled as BCl-4 and BAc-4, respectively. Additionally, the unpretreated raw material as control was also treated using the same alkali solutions and labeled as Control-4.

### Analysis methods

The SEM images of the untreated and treated *Eucalyptus* were recorded with a Hitachi 3400N scanning electron microscopy (SEM) (S-3400N, HITACHI, Japan) at acceleration voltages of 5 kV. All samples were sputtered with gold in a sputter coater (E-1010, HITACHI, Japan) prior to observation.

The Raman spectra and images of the untreated and pretreated cross sections of *Eucalyptus* cell walls were obtained on a LabRam Xplora confocal Raman microscope (Horiba Jobin–Yvon, Longjumeau, France) according to the procedure described in a previous paper [58].

The compositions of *Eucalyptus* before and after treatments were determined according to the National Renewable Energy Laboratory (NREL) standard analytical procedure [59]. The sugars were analyzed by high-performance anion exchange chromatography (HPAEC) system (Dionex ICS 3000, USA) with an AS50 autosampler, an amperometric detector, a guard PA-20 column (3 mm × 30 mm, Dionex, Sunnyvale, USA), and a Carbopac™ PA-20 column (3 mm × 150 mm, Dionex, Sunnyvale, USA) [60].

The FT-IR spectra were obtained by a Thermo Scientific Nicolet iN10 FT-IR Microscope (Thermo Nicolet Corporation, Madison, WI, USA), which was equipped with a liquid nitrogen cooled MCT detector. The spectra were recorded in the range of 4000–700 cm^−1^ at 4 cm^−1^ resolution with 128 scans per sample.

The XRD patterns of samples were performed on an XRD-6000 instrument (Shimadzu, Japan) with Ni-filtered Cu Kα radiation (*λ* = 1.54 Å) generated at 40 kV and 40 mA. The scattering angle (2*θ*) was from 5° to 40° with a step size of 0.02° every 0.5 s. The crystallinity index (CrI) was calculated based on the scattered intensity at the main peak for cellulose *I* corresponding to (002) lattice plane (*I*_002_) and the minimum intensity between the main and the secondary peaks (*I*_am_) as follows:

$$ {\text{CrI}} = \frac{{I_{002} - I_\text{am} }}{{I_{002} }} \times 100 $$

The surface topography of fiber cell walls was measured using a Nanoscope V Multimode eight atomic force microscope (AFM) (Bruker, Germany). The height images were collected in ScanAsyst mode in air at room temperature with a silicon tip. The software Nanoscope Analysis was used for image processing.

### Enzymatic hydrolysis

Enzymatic hydrolysis was performed on 2 % of substrate (w/v) in 25 mL 50 mM sodium acetate buffer (pH 4.8) using a 50 mL Erlenmeyer flask at 50 °C in a double-layer shaking incubators (ZWYR-2102C) (Shanghai, China) at 150 rpm for 72 h. Commercial cellulase (Novozyme, Beijing, China) was employed at an activity of 15 FPU/g substrate for all samples. The reactions were monitored by taking 200 μL supernatant at specific time intervals, followed by incubating the withdrawn samples in boiling water for 10 min to deactivate the enzymes, and then centrifugation for 5 min. The released glucose was analyzed by HPAEC (Dionex, ISC 3000, USA) on a CarboPac PA-100 analytical column. The experiment was performed in duplicate and the results are presented as the average values.

## Additional file

10.1186/s13068-016-0578-y The intensities of the resolved S and G Raman bands, and their ratios of the untreated and ILs pretreated *Eucalyptus*. **Figure S2.** FT-IR spectra of the control, ILs pretreated, and alkali post-treated substrates.

## Abbreviations

ILs: ionic liquids
HPAEC: high-performance anion exchange chromatography
FT-IR: Fourier transform infrared
XRD: X-ray diffraction
SEM: scanning electron microscopy
CRM: confocal Raman microscopy
AFM: atomic force microscopy
CCML: cell corner middle lamella
CML: compound middle lamella
S: secondary wall
S/G: syringyl to guaiacyl
CrI: crystallinity index
TBAA: tetrabutylammonium acetate
[Amim]Cl: 1-allyl-3-methylimidazolium chloride
[Bmim]Cl: 1-butyl-3-methylimidazolium chloride
[Bmim]OAc: 1-butyl-3-methylimidazolium acetate
[Emim]OAc: 1-ethyl-3-methylimidazolium acetate
NREL: National Renewable Energy Laboratory
