# Supplementary material for: Evaluation of the two-step treatment with ionic liquids and alkali for enhancing enzymatic hydrolysis of Eucalyptus: chemical and anatomical changes
Source: Biotechnol Biofuels. 2016 Aug 5;9:166. doi: 10.1186/s13068-016-0578-y (PMC4974680; doi:10.1186/s13068-016-0578-y)
Supplement: Supplementary file 1 — 10.1186/s13068-016-0578-y The intensities of the resolved S and G Raman bands, and their ratios of the untreated and ILs pretreated Eucalyptus. Figure S2. FT-IR spectra of the control, ILs pretreated, and alkali post-treated substrates. [file 13068_2016_578_MOESM1_ESM.docx]

**Additional file 1**

**Evaluation of the two-step treatment with ionic liquids and alkali for enhancing enzymatic hydrolysis of *Eucalyptus*: chemical and anatomical changes**

Han-Yin Li^1^

Email: [yua7v169@163.com](mailto:yua7v169@163.com)

Xue Chen^1^

Email: chenxue37121@163.com

Chen-Zhou Wang^1^

Email: wangdi142724@sina.com

Shao-Ni Sun^1,*^

Email: [sunshaoni@126.com](mailto:sunshaoni@126.com)

Run-Cang Sun^1,2,*^

Email: [rcsun3@bjfu.edu.cn](mailto:rcsun3@bjfu.edu.cn)

^1^ Beijing Key Laboratory of Lignocellulosic Chemistry, Beijing Forestry University, Beijing, 100083, China

^2^ State Key Laboratory of Pulp and Paper Engineering, South China University of Technology, Guangzhou, 510640, China

^*^Corresponding authors, ^*^Phone: +86-10-62336972.

Fax: +86-10-62336972. E-mail: rcsun3@bjfu.edu.cn.

^*^Phone: +86-10-62336592.

Fax: +86-10-62336592. E-mail: [sunshaoni@126.com](mailto:sunshaoni@126.com).

**
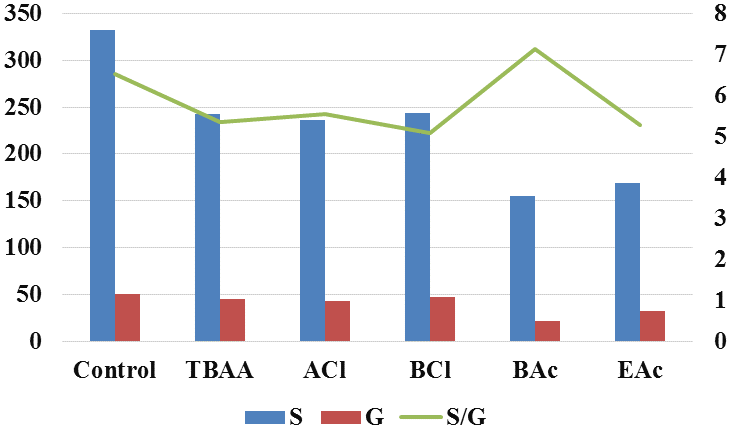
**

**Figure S1.** The intensities of the resolved S and G Raman bands, and their ratios of the untreated and ILs pretreated *Eucalyptus*.


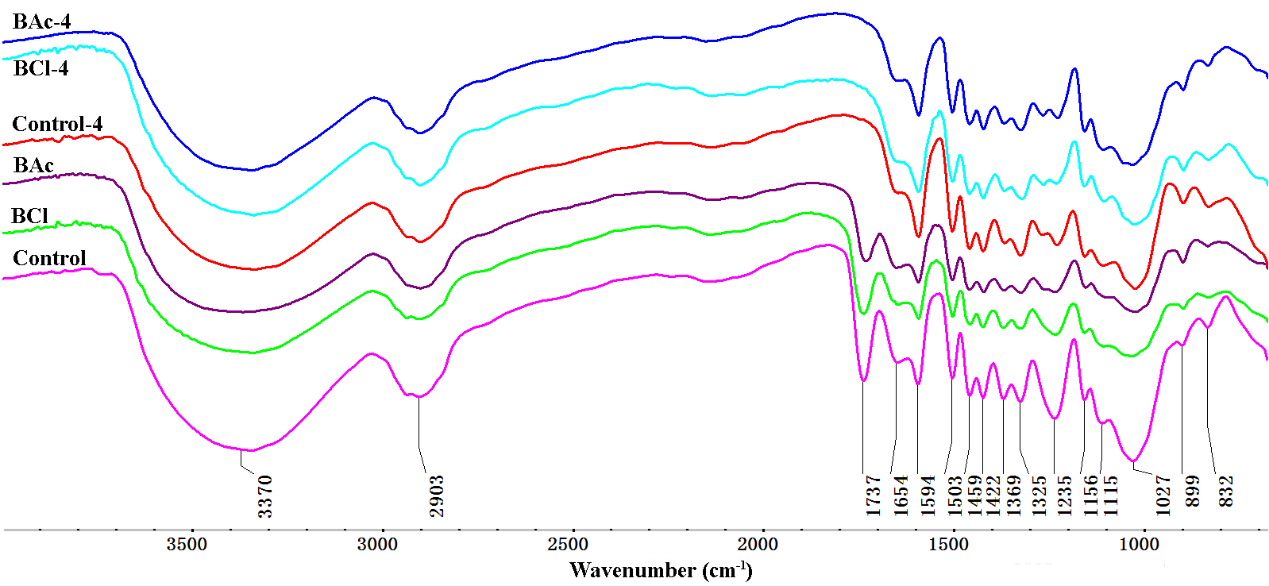


**Figure S2.** FT-IR spectra of the control, ILs pretreated, and alkali post-treated substrates.
